# An electrical probe of the phonon mean-free path spectrum

**DOI:** 10.1038/srep33571

**Published:** 2016-09-28

**Authors:** Ashok T. Ramu, Nicole I. Halaszynski, Jonathan D. Peters, Carl D. Meinhart, John E. Bowers

**Affiliations:** 1 Department of Electrical and Computer Engineering, University of California Santa Barbara, Santa Barbara, California, CA 93106, USA; 2 Department of Mechanical Engineering, University of California Santa Barbara, Santa Barbara, California, CA 93106, USA

## Abstract

Most studies of the mean-free path accumulation function (MFPAF) rely on optical techniques to probe heat transfer at length scales on the order of the phonon mean-free path. In this paper, we propose and implement a purely electrical probe of the MFPAF that relies on photo-lithographically defined heater-thermometer separation to set the length scale. An important advantage of the proposed technique is its insensitivity to the thermal interfacial impedance and its compatibility with a large array of temperature-controlled chambers that lack optical ports. Detailed analysis of the experimental data based on the enhanced Fourier law (EFL) demonstrates that heat-carrying phonons in gallium arsenide have a much wider mean-free path spectrum than originally thought.

In recent years, it has become common knowledge that phonons, the dominant carriers of heat in most solid materials including semiconductors, carry heat over a much larger range of mean-free paths (the average distance that phonons travel without scattering) than predicted by the kinetic theory^1^,^2^,^3^, resulting in a breakdown of the Fourier law over experimentally accessible length scales. The mean-free path accumulation function (MFPAF) introduced by Dames and Chen^1^ is a powerful tool in studying phonon transport in the beyond-Fourier regime, where the length scale of interest is on the order of, or less than the MFP. The MFPAF at a given mean-free path Λ is defined as the effective thermal conductivity (ETC) contributed by all phonons with mean-free paths less than or equal to Λ. The utility of the MFPAF lies in that it explains within a unified framework^2^ diverse experiments, like the transient gratings^3^, time-domain thermoreflectance (TDTR)^4^,^5^ and frequency domain thermoreflectance (FDTR)^6^ experiments, that probe heat transport on length scales comparable to the phonon mean-free path.

For bulk materials, measurements of the MFPAF have been conducted for crystalline^7^ and amorphous^8^ materials. For nanostructured materials, Yang and Dames^9^ have given a relationship to the MFPAF of bulk materials. The MFPAF of nanostructured materials has been determined by measuring length-dependent conductivity in nanowires^10^. The MFPAF has been applied to the determination of thermal properties of nanostructured materials from bulk properties^11^. The concept of MFPAF has been applied to the study of thermal interfaces as well^12^. Knowledge of the MFPAF enables nano- or micro-structuring materials for thermoelectric applications by introducing perturbations on the same scale as the MFP of phonons whose suppression is desired^13^,^14^,^15^.

We propose and implement a novel experiment to deduce the MFPAF from purely electrical measurements. We propose to excite a metal line deposited on the material to be characterized with an alternating current with frequency in the range of 200–2000 Hz–please see Fig. 1. The AC voltage developed on the heater is measured across the inner pads of the heater in order to deduce the power input given the line resistance. A temperature sensing line is deposited a short distance from the heater line. The distance between the heater and thermometer would be on the order of the mean-free path of dominant low-frequency modes (500 nm–4000 nm for bulk GaAs)^16^.

The temperature of the thermometer line is extracted by passing a direct current through its outer pads and measuring the second harmonic voltage across its inner pads (Fig. 1). This is a variant of the AC third-harmonic technique originally invented by Atalla and coworkers^17^ and popularized by Cahill and coworkers^18^ under the label “3-omega method”. We note that our “2-omega” method was originally developed to measure the thermal anisotropy of bulk materials^19^,^20^.

Prior experimental observations based on TDTR and FDTR used >10 MHz frequencies to probe the breakdown of Fourier law, thus setting the vertical length scale to ~1/100 the penetration depth in our experiment (52.2 micron at 2000 Hz), which works out to ~0.5 micron. However by using the same metal transducer as heater and thermometer, they could not reliably differentiate between the effects of interfacial resistance and the quasi-ballistic effects. This led them to laser spot-size reduction down to 2 microns in order to enhance lateral (radial) suppression, since radial suppression is independent of interfacial effects^21^.

We have taken this idea to an extreme by abandoning optical heating altogether (and concomitant diffraction limits on heater size), using a sub-micron electrical heater, and using a separate sub-micron thermometer deposited 1–2 micron away that senses the temperature solely in the radial direction. Our length scale is thus set horizontally to ~1–2 micron, similar to the (vertical) length scale of prior experiments.

The overall experimental procedure is as follows: First we conduct the standard 3-omega experiment using one of the two identical lines, heater or thermometer, and derive the bulk thermal conductivity using the slope method. The details of this experiment are described in ref. 18 and we shall not repeat it here. Next, we excite the heater with an AC current and pass a DC current through the thermometer. If the AC current has a frequency *f*, the Joule power has a frequency 2*f*, which creates temperature oscillations (TOs) at a frequency 2*f*. The TO results in a thermometer resistance oscillation at a frequency 2*f* because of the temperature coefficient of resistance of the thermometer metal line. This resistance oscillation is converted by the DC thermometer current into a voltage oscillation at a frequency 2*f* which is measured using a lock-in amplifier.

We scale the heater input power to 1 W per cm heater length. We use a frequency *f* = 2000 Hz because it is high enough to minimize finite-length effects (thus enabling a 2D analysis) and to minimize substrate effects, yet low enough for the signal strength to be adequate (please see the section “Discussion” for justification of this choice of frequency). We repeat this procedure for heater-thermometer pairs of varying separations, and plot the temperature oscillation amplitude vs. the heater-thermometer separation. We fit this graph to a two-channel phonon transport model called the enhanced Fourier law (EFL)^22^ and deduce the model parameters, and thence the MFPAF of the material being investigated. Subsequent sections provide details of the experimental procedure, and describe the results obtained for GaAs and strontium titanate.

We emphasize here that the temperature on the heater line in the 3-omega experiment is not the same as the Fourier law prediction, at least for 650 nm wide lines on GaAs–that it is not is clear from Supplementary Figure 4. However, the slope of the temperature vs. logarithmic frequency measured by 3-omega method is the same as the Fourier law prediction for the bulk thermal conductivity. On 650 nm or 2 micron wide lines, the slope gave an experimental bulk thermal conductivity of 60 W/m-K, in close proximity to the literature value of 55 W/m-K (from the NSM archive maintained by Ioffe institute, St. Petersburg, Russia). The identity of the slope of the 3-omega temperature vs. logarithmic frequency curve to the bulk Fourier law prediction, even for 650 nm narrow lines, may qualitatively be explained as follows. Since a low frequency (2000 Hz) is used, only a relatively small region around the heater, of radius on the same order of the phonon MFP (~400 nm) will have significantly suppressed effective thermal conductivity (~21.5 W/m-K–please see ‘Results’ section for these numbers). This region has a volume too small compared to the net heated region (thermal penetration depth ~52.5 micron at 2000 Hz), for heat storage due to thermal capacitance to be significant. Thus this conductivity-suppressed region appears to the heater/thermometer as a simple thermal resistor, making a frequency-independent contribution to the measured temperature. This has been quantitatively verified, as shown in Supplementary Figure 4.

However, despite the magnitude of deviations from Fourier law being considerable, 3-omega measurements on sub-micron heaters by themselves cannot be used to derive the accumulation function. The deviations are on the same order as those due to oxide and interfacial resistances, which we found to fluctuate too widely from device to device to enable unique extraction of the quasi-ballistic contribution alone. Please see Supplementary Information 6 for further details.

## Results

### Deviation of experiment from literature predictions in GaAs

The ‘2-omega’ experimental data on GaAs at room temperature is summarized in Fig. 2. Figure 2 also shows the prediction of the thermometer temperature if the accumulation function were as reported by ref. 2 for GaAs. These predictions were generated by discretizing the reported accumulation function into five channels and applying a multi-band formulation of the EFL^23^. The prediction depicted here is more accurate than that of ref. 23 because fully numerical solutions of the EFL equations were used in this work, as opposed to an approximate analytical solution. Each of the predictions falls significantly short falls significantly short of experimental findings.

For GaAs, we extract the following parameters of the MFP accumulation function from the EFL: *κ*^*LF*^ = 38.5 ± 3 W/m-K and Λ = 4 ± 0.5 micron. Here *κ*^*LF*^ and Λ are the two free parameters of the two-channel EFL, which physically represent the contribution of low-frequency (quasi-ballistic) modes to the bulk thermal conductivity, and the mean-free path at which accumulation function nears unity, respectively. The detailed equations of the EFL model are presented in Section “Methods”. From this information, we can reconstruct two points of the accumulation function, as follows^23^: (a) The conductivity of the modes obeying the Fourier law (diffusive modes), *κ*^*HF*^ = (*κ*^*bulk*^ − *κ*^*LF*^) corresponds to mean-free paths of less than 0.37 (or 1/e) times the smallest heater-thermometer separation, which is 1096/e = 403 nm in this experiment. (b) Λ corresponds to the point where the conductivity accumulates to 88% of its bulk value^23^.

### Strontium titanate as a control

The thermal conductivity of bulk STO accumulates to its bulk value within ~100 nm^24^. Therefore, our micron-scale experiment should give a null result (no deviation from the Fourier law within experimental error). That this is indeed the case is evident from Fig. 3. The deviations are small relative to the temperature oscillation amplitude (4–7 K), and even negative deviation is seen for certain separations. Error bars in the measured 2-omega temperature oscillation amplitude for STO with respect to the nominal (average) value are also shown; we estimate that known sources of error total between −1% and +2%. Please see Supplementary Information 2 for the breakdown of total error by source.

### The new proposed accumulation function for GaAs

In Fig. 4, we plot the accumulation function of GaAs corresponding to our extracted parameter set (please see the section “Results”) and normalized to the bulk conductivity (60 W/m-K) together with the accumulation function reported by ref. 2 and the first-principles calculations of ref.  25. It is seen that they differ widely.

The seminal work of Freedman *et al*.^2^ was the first serious attempt at MFPAF extraction from thermoreflectance data and identification of a unified MFP-resolved spectrum describing several crystalline materials through scaling of the independent variable. However, extraction of such wide swathes of the MFPAF from frequency-domain thermoreflectance data as shown in Fig. 4 was earlier criticized by some of the authors on grounds that the raw experimental data could be fit equally well using the compact two-parameter set provided by the EFL (please see ref. 26). The point was also made that the underlying assumption (due to Koh and Cahill ref. 27) that phonons of MFP greater than the thermal penetration depth are entirely lost to the measurement was *prima facie* incorrect. Other criticism of ref.  2 includes the argument of ref.  28 that the diffusion equation was incorrectly used. The agreement of ref.  2 with the first-principles calculations of ref.  25 should serve as a precaution against using these calculations in the stead of actual experiment.

## Discussion

The 50 nm oxide layer (deposited for electrical isolation of the metal lines from the unintentionally conducting substrates) as well as the effects of the finite thickness of the heater line have been ignored in the analysis. Here we seek to rule out the possibility that (a) the oxide layer may cause a thermal impedance and an accompanying temperature drop between the thermometer and the substrate, thus making the measured temperature greater than the actual temperature of the material surface (b) the finite thickness of the heater line may cause some heat storage in the heater, so that the power fluxed into the material may not equal the power input, and (c) the finite thickness of the thermometer line may result in a thermal impedance between it and the material under test.

Figure 5 shows Fourier law simulations including as well as excluding all three effects, assuming 500 nm wide, 1.2 micron thick metal lines separated by 1000 nm (center-center) deposited on 50 nm SiO_2_ deposited on GaAs. The thermal conductivity of the oxide is taken to be 0.67 W/m-K as estimated by fitting to 3-omega data on GaAs. This deviates from the nominal oxide thermal conductivity of 1.1 W/m-K in that it includes the boundary thermal impedance between the heater and oxide, and between oxide and GaAs. The thermal conductivity of the metal line is taken as 205 W/m-K, in accord with the accepted value for Al. Where all three effects (a–c) above are excluded, a heat-flux boundary condition is used, which implies that all the input power is fluxed into the material, with no storage in the heater. It is clear from Fig. 5 that all three effects mentioned above are negligible, altogether causing a net error of ~0.2% in the temperature oscillation amplitude.

The finite length of the heater and thermometer lines (300 micron) may cause three-dimensional edge effects neglected in our 2-D analysis. In Fig. 6 we plot a sample experimental dataset together with 2D and 3D simulations of the Fourier law as well as the EFL. The metal lines on 50 nm SiO_2_ on GaAs are 652 nm wide and ~1.2 micron thick, separated center-to-center by 1094 nm. The parameters used for 2D and 3D EFL simulations are *κ*^*LF*^ = 38.5 W/m-K and Λ = 4 micron, which gives the best overall fit to all collected data. Please see Table 1 for the remaining parameters.

We see that 3D effects are negligible at the frequency used throughout in the analysis, 2000 Hz. However, 3D effects are by no means negligible at 200 Hz. (Below 200 Hz, effects due to the finite thickness of the substrate become important, and therefore simulations are not carried out at lower frequencies). The thermal penetration depth at this frequency in GaAs is 52.2 micron, and this decreases as the inverse square-root of the frequency. Thus operating at higher frequencies would make the experiment even more sensitive to quasi-ballistic effects, which dominate the temperature profile within a mean-free path of the heater surface. This however has to be traded off with decreasing temperature signal and attendant degradation of the signal-to-noise voltage ratio, which we estimate at an excellent 120 dB at 2000 Hz.

We further conjecture the possibility of using frequency-dependent 2-omega data to refine the accumulation function to yield a series of small steps instead of two points (Fig. 4), since it is seen that the 200 Hz data could not be fit adequately by 3D simulations that utilized the EFL parameter set derived at 2000 Hz. However, we were dissuaded from doing so owing to the extreme computational expense incurred by 3D simulations, especially by the iterative optimization routine used to derive best-fit parameters from the experimental data.

The overall error in the measured 2-omega temperature oscillation amplitude for GaAs with respect to the nominal (average) value is −1.3% to +3.3%. We have included all known sources of error in this analysis. Since the repeatability of the experiment (Fig. 2) is ±5%, we conclude that the bulk of the repeatability bars are due to as-yet undetermined sources of error. However, the final result (MFPAF of GaAs) is not overly sensitive to these errors, as evidenced by the tolerable error bars on Fig. 4. We have broken down known errors down by source in the Supplementary Information 2.

We have measured the bulk thermal conductivity of GaAs as 60 W/m-K. This compares well to the accepted value of 55 W/m-K (from NSM archive maintained by Ioffe institute, St. Petersburg, Russia). If the latter value were used in the modeling, we would extract *κ*^*LF*^ = 36 W/m-K and Λ = 2.8 micron. We have shown this as part of the error bar in the final result for the accumulation function of GaAs in Fig. 4.

## Methods

### Theoretical analysis

We analyze the experimental data in the context of the two-channel enhanced Fourier law (EFL) developed earlier^22^,^29^, which accounts for quasi-ballistic effects by dividing the phonon population into two parts (a) a high heat-capacity, high phonon frequency channel that obeys the Fourier law, and (b) a low heat-capacity, low phonon frequency channel that carries heat quasi-ballistically. The limitations of the EFL model are discussed in the Supplementary Information 3.

We desire the temperature profile on a thermometer of width *w*_*t*_ separated from a heater of width *w*_*h*_ by a center-to-center distance of *D*. All variables (excluding parameters) are assumed to have an implicit time dependence *e*^−*j*2*ωt*^. First we state the enhanced Fourier law (EFL) in three dimensions as derived in ref. 22, ignoring circulation of the heat-flux:


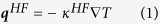



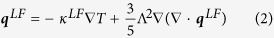


***q***^*HF*^ is the high-frequency (HF) heat-flux that follows the Fourier law, *κ*^*HF*^ is the corresponding thermal conductivity, ***q***^*LF*^ is the heat-flux in the low-frequency (LF) quasi-ballistic mode, Λ is the mean-free path of the LF mode, *κ*^*LF*^ is the kinetic theory value of the LF modal thermal conductivity, and *T* is the temperature. We note that *κ*^*HF*^ + *κ*^*LF*^ = *κ*^*bulk*^ (the bulk thermal conductivity, a known quantity) so that the only independent parameters in this model are *κ*^*LF*^ and Λ.

Next we state the law of energy conservation:


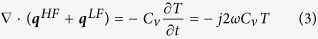


*ω* is the angular frequency of the heater current and *C*_*v*_ is the heat capacity of all phonon modes (HF and LF).

The boundary conditions at the heater are given by,


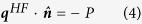



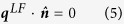


Here *P* is the power fluxed by the heater per unit area, calculated using a total power assumed henceforth to be fixed at 1 W per cm heater length. As mentioned in the introduction, all experimental data is scaled to this input power before presenting in this paper. Quantities ***q***^*HF*^, ***q***^*LF*^and *T* re assumed to go to zero at infinite distance (which is set to the substrate thickness, 500 micron).

From inspection of the analytical form of these equations, it can be seen that they represent a system of linear partial differential equations. Therefore, even though the equations contain the non-Fourier term depending on 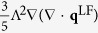, the superposition principle holds. In particular, sinusoidal inputs (in our case, injected through the boundary conditions) produce sinusoidal outputs, once the initial temperature transients decay down. This justifies our calculations in this section. Please see Supplementary Information 4, where we will confirm with a simple 1D model that:

- The initial transients indeed decay down, and that
- Both the −*κ*^*LF*^∇*T* and the 
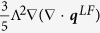
 terms do indeed contribute to the heat-flux ***q***^*LF*^. If the first term −*κ*^*LF*^∇*T* alone contributed, we would recover the Fourier law temperature. We show that we do not.

**Figure 1:**
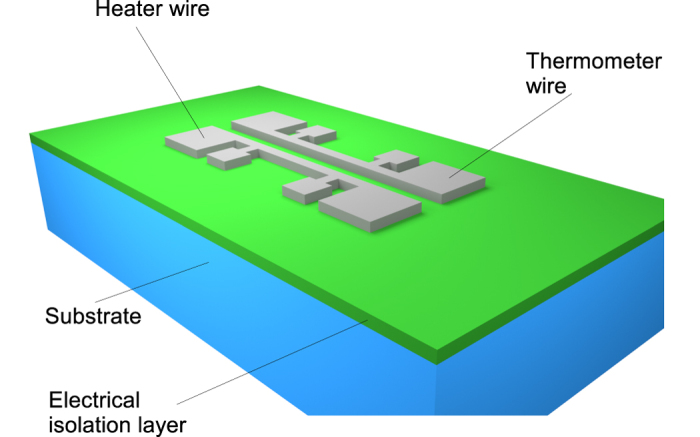
The ‘2-omega’ method. Schematic of proposed experiment to investigate non-Fourier heat transfer.

**Figure 2:**
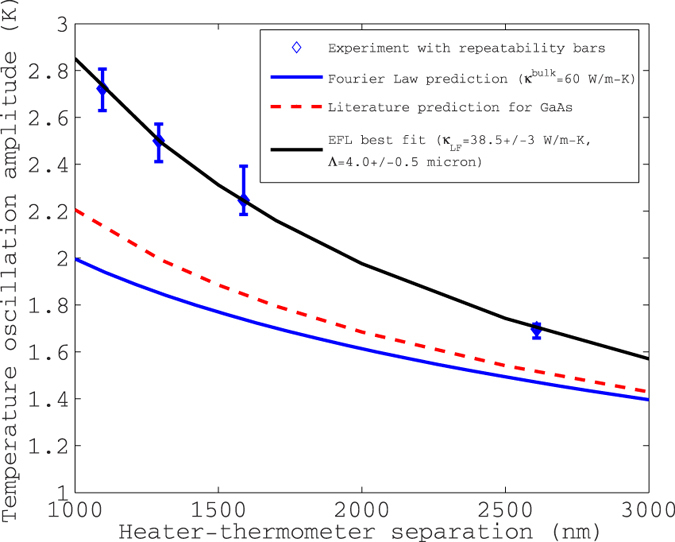
Data versus literature and Fourier law predictions on GaAs. Experimental temperature oscillation amplitude and the Fourier law prediction for GaAs at 300 K, plotted as a function of heater-thermometer center-to-center separation. The data is fit to the enhanced Fourier law (EFL) and the parameters of the accumulation function are extracted as *κ*^*LF*^ = 38.5 ± 3 W/m-K and *Λ* = 4 ± 0.5 micron. The uncertainty in parameter values reflects the repeatability bars shown in the figure. Also shown is the predicted curve using the accumulation function reported in the literature^2^,^25^.

**Figure 3:**
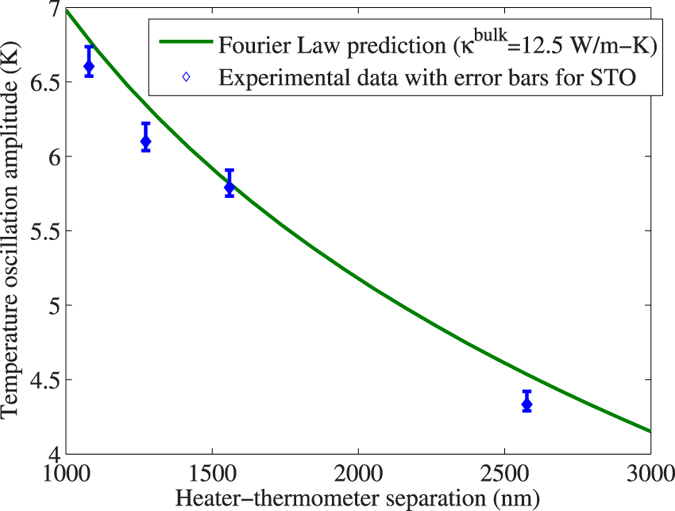
Null result on strontium titanate control. Experimental temperature oscillation amplitude and the Fourier law prediction for strontium titanate at 300 K, plotted as a function of heater-thermometer center-to-center separation. The differences are small relative to the temperature oscillation amplitude, the latter being on the order of 4–7 K.

Equations (1, 2, 3, 4, 5) are solved numerically for the geometry of Fig. 1 using the finite-element method implementation COMSOL®. The two-channel EFL simulation is noted at each of four heater-thermometer separations *D*, and parameters *κ*^*LF*^ and Λ are varied iteratively until the simulation best matches the experimental data in the least mean-square sense. COMSOL®’s optimization solver is used for this purpose.

**Figure 4:**
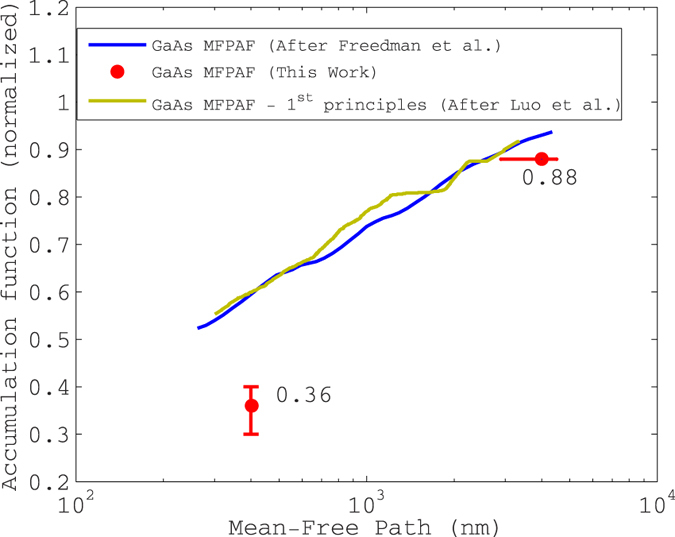
Phonon mean-free path accumulation function for GaAs at room temperature. The current work shows that only 36% of the bulk thermal conductivity may be attributed to phonons of MFP less than 400 nm, whereas Freedman *et al*.^2^ estimates this at 60%. After Freedman *et al*.^2^. The first principles calculations are after ref. 25.

**Figure 5:**
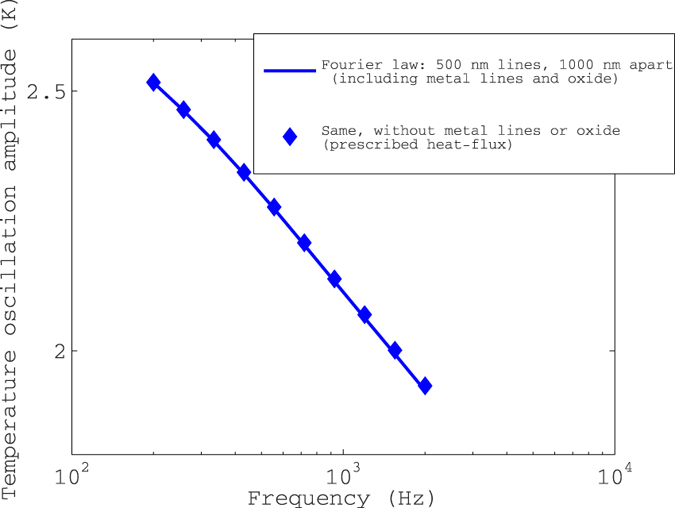
Negligible effect of boundary thermal impedances. Fourier law simulations including and excluding the finite thickness of the heater and thermometer lines, as well as the effect of the 50 nm SiO_2_ layer. the effect of the 50 nm SiO_2_ layer.

**Figure 6:**
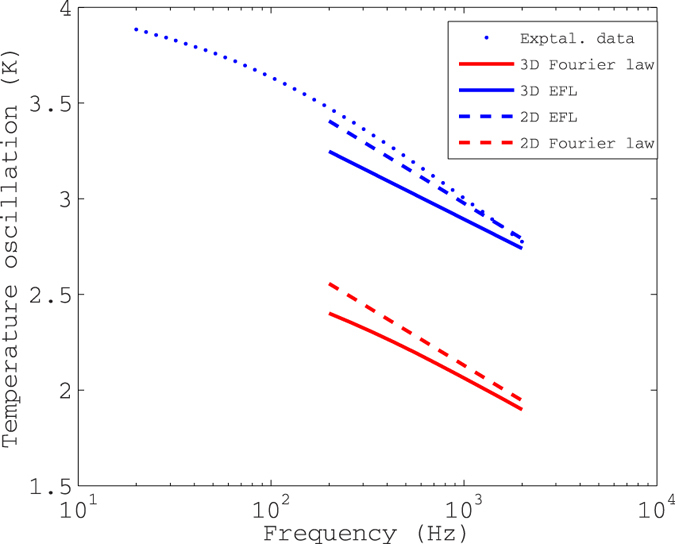
Sample experimental data. Sample experimental data together with 2D and 3D analyses using both the Fourier law and the enhanced Fourier law (EFL). Please see text (“Discussions”) for details.

Once *κ*^*LF*^ and Λ are known, an approximation for the mean-free path accumulation function may be obtained as shown in Fig. 4. We neglect the thin electrical isolation layer as well as the finite thickness of the heater in our analysis of the ‘2-omega’ experiment; we have justified these approximations in the “Discussions” section.

### Experimental methodology

On the GaAs substrate, 50 nm silicon dioxide is deposited using plasma-enhanced chemical vapor deposition. Aluminium heater-thermometer pairs with each line of length 300 microns, width varying as 500nm, 1000 nm, and 2000 nm, and thickness of 1.2 microns, are fabricated on top of the oxide on GaAs, with a 23 nm titanium film acting as an adhesion layer. Thus the oxide layer electrically isolates the heater/thermometer pair from the unintentionally conducting GaAs substrate. An array of devices is made with heater-thermometer separations ranging over 1000, 1200, 1500 and 2500 nm. After initial 3-omega experiments to yield the bulk thermal conductivity, the heater/thermometer widths are fixed at 500 nm (nominally) for the 2-omega experiment, and their separation *D* alone is varied over the range mentioned above.

On strontium titanate (STO) substrate, the fabrication process is slightly different. The 50 nm oxide layer is deposited using radio-frequency sputtering, and the metallization consists of Ti/Au/Ti/Au 20/10/20/~500 nm. The rest of the dimensions are nominally the same. Process details are provided under Supplementary Materials 1.

The dimensions mentioned above are nominal, as laid out on the photolithography mask. Actual dimensions were measured using scanning electron microscopy (SEM) on each device that was probed, and the measured values were used in analyses. The width of the nominally 500 nm wide lines was actually 650 nm on an average, with deviations of +/−40 nm depending on the device. The sensitivity of the modeling to the heater/thermometer widths is very small; for example varying the heater and thermometer widths from 610 nm to 690 nm resulted in only a <0.5% change in the Fourier law result for the temperature of the thermometer on GaAs, for a constant heater-thermometer center-to-center separation of 1096 nm. Additionally, our measurements (please see Supplementary Information 5 for 2-omega data for all 15 devices on GaAs) show no correlation of the measured temperature to the heater width. Thus we can plot data points collected on devices with different heater and thermometer widths on the same graph. Earlier literature^21^,^30^,^31^ has examined the heater size dependence of the effective thermal conductivity. Although those papers reported on the order of 30–50% change to the effective thermal conductivity, this was for a spot-size ranging from 1 micron to 10 micron. It can be seen that our heater width fluctuations are much smaller, and our findings are therefore not inconsistent with these studies. The mean measured center-center heater-thermometer separations were 1096, 1292, 1566 and 2609 nm. These separations showed much less spread from device to device (only +/−10 nm) than the individual line widths.

For implementation of the “2-omega” method, a 5 V-rms sinusoidal waveform generator feeds the outer pads of the heater through a 100 Ohm resistor. A 10 V DC source feeds the outer pads of the thermometer through a 1 kilo-Ohm resistor. The resistors are included to reduce the noise introduced into the circuit by the sources. The nominally 500 nm wide lines have a resistance of 20–40 Ohm. The voltage across the inner pads of the heater is noted by a lock-in amplifier, in order both to monitor the power input as well as to provide a reference phase for detection. The second harmonic of the voltage across the inner pads of the thermometer is noted by the lock-in amplifier, and converted into a temperature oscillation amplitude. The component of the thermometer second harmonic voltage in phase with the heater power alone is used, since the out-of-phase signal is much weaker. Some typical values for various voltages are: 0.8 V-rms for the AC voltage across the inner pads of the heater, 0.3 V for the DC voltage across the inner pads of the thermometer, and 1.2 millivolt-rms and 200 microvolts-rms for the second harmonic voltage across the inner pads of the thermometer in phase and out of phase with the heater power respectively, at a frequency of 2000 Hz. The second harmonic voltage across the inner pads of the thermometer is converted into a temperature oscillation amplitude using the relation^20^:


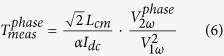


Here *L*_*cm*_ is the length of the heater, *α* is the temperature coefficient of resistance of the thermometer, *I*_*dc*_ is the DC current through the thermometer, 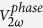 is the second harmonic voltage across the inner pads of the thermometer in phase with the heater power, and *V*_1*ω*_ is the AC voltage across the inner pads of the heater line. 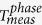 has units of K/(Watt/m). If all voltages are measured in volts, *I*_*dc*_ in amperes, and *L*_*cm*_ in centimeters, then 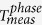 corresponds to the temperature in K assuming an input power of 1 W per cm heater length. Throughout the paper, we have scaled all experiment and simulations to a heater power of 1 Watt per cm heater length.

Three to five devices (i.e. heater-thermometer pairs) were measured for each value of the separation in order to establish the repeatability of the experimental technique, and the results were fit to the two-channel enhanced Fourier law (EFL) implemented in COMSOL®, using COMSOL®’s optimization solver. Input parameters to the model are summarized in Table 1.

## Conclusions

In conclusion, a novel purely electrical probe of the phonon mean-free path accumulation function has been proposed with notable advantages over traditional optical methods, namely insensitivity to interfacial thermal impedances and compatibility with temperature-controlled chambers that lack optical ports. Significant deviations from the Fourier law have been noted for this experiment when conducted on bulk GaAs, and the MFP spectrum of GaAs is seen to be much wider than previously reported. 2-omega experiments analyzed using the enhanced Fourier law suggest that phonons in GaAs with MFP up to 400 nm carry just 36% of the bulk thermal conductivity. This experiment being sensitive to the accumulation function in the region spanning several hundreds of nm to several microns, forms a useful tool along with transient grating and frequency-domain thermoreflectance experiments in mapping out the accumulation function of bulk matter. The accurate delineation of the phonon mean-free path spectrum is vital for thermoelectric applications that depend on sustaining large temperature gradients.

The current dimensions of line width, pitch, and metal thickness using negative sloped liftoff is close to the limit of what we can reasonably reproduce in our current i-line lithography process. As future outlook, a change to 248 nm DUV (deep ultraviolet) or electronic beam lithography (EBL) would be necessary to achieve smaller linewidths. We are examining alternate processes such as selective plating or inductively-coupled plasma (ICP) dry etch, which are promising for 50 nm wide lines with a pitch of 400 nm. In addition, extending the heating frequency to 1 MHz will significantly improve depth resolution.

The main limitation of our technique is that it trades off simple instrumentation and simple data analysis for a complex fabrication process (when compared to optical techniques). However this limitation actually derives from an advantage over optical techniques–while it may be challenging to achieve 50 nm wide lines spaced 400 nm, it is physically impossible to focus currently available coherent laser sources (wavelength > = 126 nm) on to 50 nm spot sizes due to the diffraction limit. There has been at least one effort^32^ to make the effective heater size 50 nm with a 60–100 micron spot-size laser, but the fabrication process becomes substantially more complicated than ours. Also, this effort again heats the sample and measures the temperature over the same thermal reservoir, which requires additional modelling to extract out interfacial effects.

## Additional Information

**How to cite this article**: Ramu, A. T. *et al*. An electrical probe of the phonon mean-free path spectrum. *Sci. Rep.* **6**, 33571; doi: 10.1038/srep33571 (2016).

## Supplementary Material

Supplementary Information

## Figures and Tables

**Table 1:** Material parameters used in the modeling of GaAs and STO substrates.

Parameter	Value	Source
*κ*^*bulk*^for GaAs	60 W/m-K	‘3-omega’ measurement
*κ*^*bulk*^for STO	12.5 W/m-K	‘3-omega’ measurement
Heat capacity *C*_*v*_, GaAs	1.76 × 10^6 ^J/m^3^-K	NSM archive, Ioffe Institute, Ru.
Heat capacity *C*_*v*_, STO	2.75 × 10^6 ^J/m^3^-K	ref. 33
*α* for Ti/Al 23/1200 nm	3.7 × 10^−3 ^K^-1^	I-V-T measurements
*α* for Ti/Au/Ti/Au 20/10/20/500 nm	3.3 × 10^−3 ^K^−1^	I-V-T measurements
