# Supplementary material for: An electrical probe of the phonon mean-free path spectrum
Source: Sci Rep. 2016 Sep 28;6:33571. doi: 10.1038/srep33571 (PMC5039410; doi:10.1038/srep33571)
Supplement: Supplementary Information [file srep33571-s1.pdf]

An electrical probe of the phonon mean-free path spectrum

Ashok T. Ramu[1], Nicole I. Halaszynski[1], Jonathan D. Peters[1], Carl D. Meinhart[2] and John E. Bowers[1]

1. Department of Electrical and Computer Engineering, University of California Santa Barbara, Santa Barbara, California, CA-93106, USA.
2. Department of Mechanical Engineering, University of California Santa Barbara, Santa Barbara, California, CA-93106, USA.

1. Process for fabricating devices for testing

Here we describe the device fabrication process in detail in order to facilitate replication of our results.

(a) On GaAs wafers:

50 nm SiO_2 was deposited by plasma-enhanced chemical vapor deposition (PECVD) at 250 C on a clean, double-side polished 2-inch diameter unintentionally doped GaAs wafer. The wafer was coated with photoresist NR9-1000 from Futurrex®, and spread to a uniform thickness of 1300 nm by spinning at 2000 rpm. After a 135 C bake for 3 min, it was exposed to UV radiation under a photo-mask for 0.92 sec in a GCA Auto-stepper. A post-develop bake was performed at 100C for 2 min, followed by resist development by exposure to developer MF726 from MicroChemicals® for 20 sec. The development was completed with an O_2 plasma exposure for 30 sec to de-scum developed areas. A Ti adhesion layer 23 nm thick was deposited on the developed wafer, followed by a 1200 nm Al layer, both deposited by electron-beam (e-beam) evaporation. Finally, the metal was lifted off from undeveloped areas using 1165 stripper from MicroPosit®.

(b) On strontium titanate (STO) wafers:

An STO wafer 30 mm in diameter was cleaned and 50 nm SiO_2 was deposited by radio-frequency sputtering, since the wafer could not withstand the 250 C temperature of the PECVD chamber. The deposition conditions were as follows: RF power = 250W, DC Bias = 150 Volts, O_2 flow rate = 2.5 sccm, Ar flow rate = 25 sccm, silicon target as the source of silicon, pressure = 3 mTorr, temperature = 20 C. Since the STO wafer was transparent, the resist deposited directly on SiO_2 failed to develop, and the wafer had to be rendered opaque to enable resist deposition. This was accomplished by deposition of 20/10 nm Ti/Au layer on the oxide by e-beam evaporation. The resist deposition and patterning proceeded exactly as for the GaAs wafer. This was followed by e-beam metal deposition (Ti/Au 20/600 nm) and lift-off in 1165 stripper. This metal layer served both as a hard mask for removing the original Ti/Au layer wherever it was not needed, as well as the heater/thermometer metallization. The original Ti/Au 20/10 nm layer was etched away using inductively-coupled plasma (ICP) etch for 90 sec. The ICP etch conditions were as follows: Cl_2 flow rate = 44 sccm, Ar flow rate = 20 sccm, pressure = 1.3 Pa, RF power = 200 W, DC Bias = 50 V. Etch rate calibrations showed that the Ti etch rate = 40 nm/min, Au etch rate = 20 nm/min, SiO_2 etch rate= 40 nm/min and SrTiO3 etch rate = 4nm/min. Thus the 90 sec ICP etch removed the Ti and Au layers between the heater and thermometer lines with minimal to no invasion of the STO substrate.

2. Error analysis:

a. Errors in the measured 2-omega temperature oscillation amplitude for GaAs with respect to the nominal (average) value: Overall error: -1.3% to +3.3%. Breakdown by source of error:

i. Voltage measurement error: ±0.1%, going by fluctuations after stabilization of readings. This translates to an error in quoted temperature of ±0.3%.
ii. Heater width fluctuation of 650±40 nm: ±0.25% (this is the worst case – it drops to ±0.05% at 2609 nm heater-thermometer separation)
iii. Fluctuations in heater-thermometer center-center separation of ±10 nm: ±0.5%
iv. Worst case error due to fluctuations in the oxide width and interfacial impedance: ±0.25%
v. Correcting for 3D heat-flow instead of assumed 2D heat-flow: +2% (we overestimate temperature during 2D simulations, and to offset this we must subtract 0-2% from the measured data depending on the separation, widths etc.)

b. Errors in the measured 2-omega temperature oscillation amplitude for $SrTiO_3$ with respect to the nominal (average) value: Overall: -1% to +2%. Breakdown by source of error:
i. Voltage measurement error: ±0.1%, going by fluctuations after stabilization of readings. This translates to an error in quoted temperature of ±0.3%.
ii. Heater width fluctuation of 525±40 nm: ±0.125%
iii. Fluctuations in heater-thermometer center-center separation of ±10 nm: ±0.36%
iv. Worst case error due to fluctuations in the oxide width and interfacial impedance: ±0.2%
v. Correcting for 3D heat-flow instead of assumed 2D heat-flow: +1% (we overestimate temperature during 2D simulations, and to offset this we must subtract 0% to 1% from the measured data depending on the separation, widths etc.)

The errors in the experimental data described above are of course reflected as uncertainties in the derived parameters of the accumulation function. κ^{HF} and Λ are the two parameters of the MFPAF that we seek to determine from experiment. We use the larger repeatability bars instead of the above errors to determine that κ^{HF}=38.5±3 W/m-K and Λ =4±0.5 micron.

However one more source of error enters here: the EFL model requires the bulk thermal conductivity as input. Although we measure this at 60 W/m-K for GaAs from 3-omega experiments, the literature value is 55 W/m-K. Assuming a fluctuation between 55 and 60 W/m-K gives us the final bound on the MFPAF parameters as κ^{LF}=38.5±4 W/m-K and Λ=2.9-4.5 micron. This is plotted in Fig. 4 of the manuscript showing the literature MFPAF vs this work.

3. Limitations of the EFL model

In a 1D ballistic channel with two thermal reservoirs, the ballistic resistance comes from thermalizing at the contacts. This leads us to address the question: what is the origin of such a resistance in our experimental geometry?

To answer this, we have to digress somewhat and discuss why the thermal conductivity is experimentally *suppressed* due to quasi-ballistic effects, and then outline what would happen if the reason for suppression were circumvented.

We define κ^{LF} as the portion of the *bulk* thermal conductivity carrier by low-frequency (LF) phonons (typically < 1 THz), if they were to propagate diffusively, and as such it is finite by construction. The term $\frac{3}{5}\Lambda^2\nabla(\nabla\cdot\boldsymbol{q}^{LF})$ in the differential equation for $\boldsymbol{q}^{LF}$ affects what the actual conductance of the LF modes is. Generally, κ^{LF} is suppressed in experiment. Although ballistic effects should on the face of it improve thermal transport, ballistic modes have a small density of states and therefore coupling to these modes is very small across heating interfaces. Thus these modes remain populated only by the Bose function at ambient temperature, and are random in k-space, carrying no net heat. They can only be

activated (for purposes of heat transport) by recombining (scattering) with the reservoir (HF) phonons. The $\Lambda^2\nabla(\nabla \cdot \boldsymbol{q}^{LF})$ term together with energy conservation captures this recombination rather precisely. However by the time they recombine, the LF phonons would have travelled so deep that temperature gradients would be too small for the $\Lambda^2\nabla(\nabla \cdot \boldsymbol{q}^{LF})$ to be active any longer, at least for the low frequencies assumed in this paper. The net effect is a suppressed thermal conductivity.

However, if we were to assume that the LF modes were populated directly by a DC heater and there were no HF modes (which is congruent to the example of 1D heat transport by ballistic phonons between thermal reservoirs) we arrive at an effective thermal conductivity pursuant to the EFL model of κ^{LF}! This is obviously incorrect – the maximum conductance in this example should be given by the phonon radiation limit and the contact ballistic conductance, and the conductivity should be given by this conductance times the distance between reservoirs – a function of length.

The inconsistency is due to the underlying assumption of the EFL model (please see derivation in [1]) that the LF mode distribution function strives to equilibrate to the local Bose function at a temperature set by the HF modes that are assumed to possess a large heat capacity and hence a well-defined local temperature. (We have carefully avoided defining a 'temperature' for quasi-ballistic modes). Thus it appeals to the concept of a local temperature and therefore presupposes a local thermal reservoir. In the above example, the system is so far out of thermal equilibrium that there is no notion of local temperature except at the contacts – there are no 'HF' modes.

The brief answer to the question is that the origin of a finite contribution of the LF modes is the inability of our formalism to deal with truly ballistic transport. However going back to the original experiment, we note the following:

(a) The LF modes emitted by the heater are far from the radiation limit, which assumes perfect emissivity for the heater. In practice, the heater-GaAs interface back-scatters phonons, and the acoustic mismatch model is more realistic for low-frequency phonons. More importantly, the heat-capacity of the LF modes is on the order of 10^4 J/m^3-K, about 1% of the total. We generously estimate the interfacial thermal conductance of LF modes as a mere 11 MW/m^2-K even with nearly perfect transmission. Thus recombination with HF modes is the predominant source of LF-mode thermal conduction.

(b) Maznev *et al.* [2] have discussed the suppression function for the transient grating experiment, and our EFL model based on LF-HF recombination has predicted the correct suppression function as documented in prior work ([3]).

(c) As an aside, the EFL is still beyond the Fourier law, because it *does not* assume that the actual LF mode distribution function is Bose with the local (spatially varying) temperature – it adds correction terms to accommodate more strongly peaked distribution functions.

4. Linearity of the EFL equations

Let us examine our equations of quasi-ballistic transport:

$$\boldsymbol{q}^{HF} = -\kappa^{HF}\nabla T \tag{1}$$

$$\boldsymbol{q}^{LF} = -\kappa^{LF}\nabla T + \frac{3}{5}\Lambda^2\nabla(\nabla \cdot \boldsymbol{q}^{LF}) \tag{2}$$

$$\nabla \cdot (\boldsymbol{q}^{HF} + \boldsymbol{q}^{LF}) = -C_v\frac{\partial T}{\partial t} \tag{3}$$

From inspection of the analytical form of these equations, it can be seen that they represent a system of linear partial differential equations. Therefore, even though the equations contain the non-Fourier term

depending on $\frac{3}{5}\Lambda^2\nabla(\nabla\cdot\mathbf{q}^{\mathrm{LF}})$, the superposition principle holds. In particular, sinusoidal inputs (in our case, injected through the boundary conditions) produce sinusoidal outputs - once the initial temperature transients decay down. This justifies our calculations in the 'Methods' Section.

We will confirm with a simple model that

(a) The initial transients indeed decay down, and that

(b) Both the $-\kappa^{LF}\nabla T$ and the $\frac{3}{5}\Lambda^2\nabla(\nabla\cdot\boldsymbol{q}^{LF})$ terms do indeed contribute to the heat-flux $\boldsymbol{q}^{LF}$. If the first term $-\kappa^{LF}\nabla T$ alone contributed, we would recover the Fourier law temperature. We show that we do not.

Our model consists of a 1D bar 500 micron long, with periodic heating at x=0 with amplitude *P*=500 W/m^2 and frequency 2048 Hz. The initial conditions are -5 mK at x=0 and +5 mK at x=500 micron, linearly interpolated inside the bar. We solve Eqs. 1-3 for this system with the same boundary conditions as in the paper, namely: The boundary conditions at the heater are given by,

$$\boldsymbol{q}^{HF}\cdot\hat{\boldsymbol{n}} = -P \qquad (4)$$

$$\boldsymbol{q}^{LF}\cdot\hat{\boldsymbol{n}} = 0 \qquad (5)$$

All quantities $\boldsymbol{q}^{HF}$, $\boldsymbol{q}^{LF}$and T are assumed to go to zero at infinite distance (which is set to the substrate thickness, 500 micron)

The rest of the parameters are κ^{HF}=22 W/m-K, κ^{LF}=38 W/m-K, Λ = 4 micron, C_v=1.75X10^6 J/m^3-K. The finite-difference method was used in the computation.

(a) Supplementary Fig. 1 shows that the initial temperature transients do indeed decay leading to a pure sinusoid.
(b) Supplementary Fig. 2 shows that $\boldsymbol{q}^{LF}$ is non-zero.
(c) More importantly, supplementary Fig. 3 shows significant deviations from Fourier law. This demonstrates that the $\frac{3}{5}\Lambda^2\nabla(\nabla\cdot\mathbf{q}^{\mathrm{LF}})$ term is active as well.

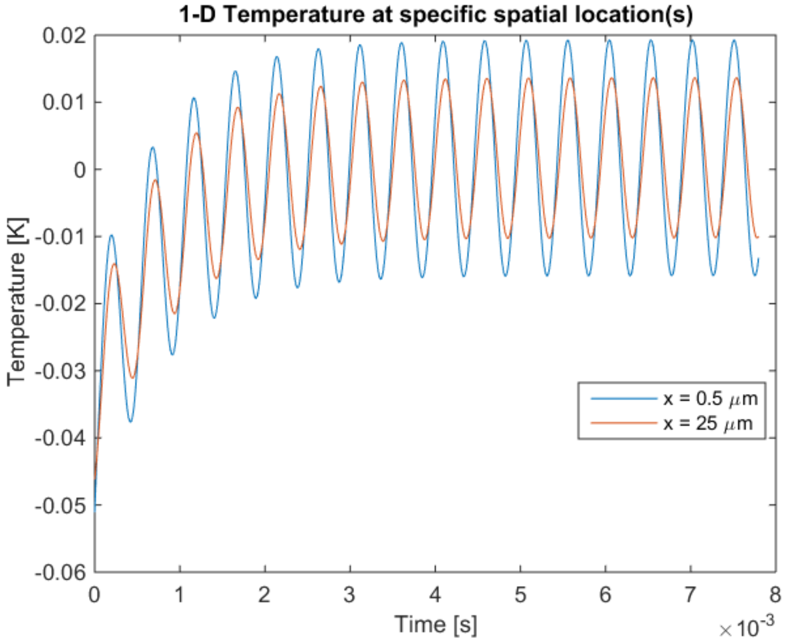

Supplementary Figure 1: Initial temperature transients decay to zero.

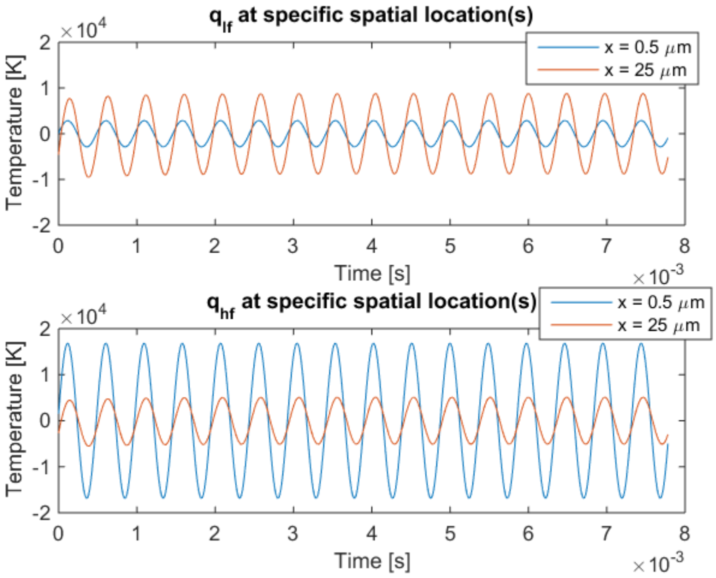

Supplementary Figure 2: $\boldsymbol{q}^{LF}$ is a substantial contribution to the overall heat-flux

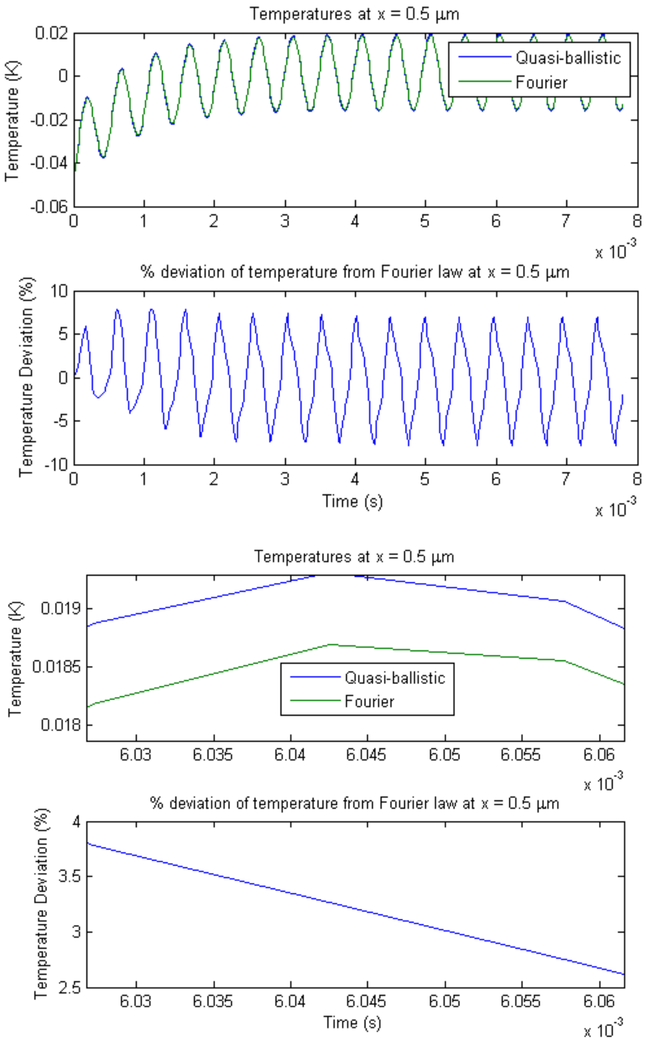

Supplementary Figure 3: Simulations of temperature vs. time with and without the non-Fourier $\frac{3}{5}\Lambda^2\nabla(\nabla\cdot\boldsymbol{q}^{LF})$ *term. Deviations from Fourier law are significant, as the zoomed plots show.*

5. Experimental 2-omega data on all measured devices, with corresponding SEM line widths and separations
    a. GaAs substrate

Device ID	Heater-Thermometer (c-c) separation (nm)	Heater Width (nm)	Fourier Law at 2KHz (K)	Scaled Expt. Temp. at 2KHz (K)	Deviation from Fourier Law at 2KHz (K)
AD15	2603	678	1.4723	1.66	0.1877
AD23	1584	667	1.7405	2.104	0.3635
BA45	1094	655	1.9458	2.776	0.8302
BA53	1301	661	1.8488	2.57	0.7212
BC15	2620	684	1.4688	1.717	0.2482
BC23	1593	667	1.7374	2.347	0.6096
BC45	1097	643	1.9435	2.642	0.6985
BC53	1301	625	1.8474	2.407	0.5596
BD53	1274	637	1.8595	2.524	0.6645
CA45	1097	643	1.9435	2.764	0.8205
CB15	2605	625	1.4713	1.717	0.2457
CB23	1584	625	1.7395	2.232	0.4925
DA45	1096	637	1.9437	2.806	0.8623
DC23	1590	649	1.738	2.306	0.568
DC45	1095	614	1.943	2.628	0.685

    b. Strontium titanate substrate

Device ID	Heater-Thermometer Separation (nm)	Heater/Thermometer Width (nm)	Expt. at 2KHz(K)	Fourier Law (K)	Deviation (K)
AA45	1079	497	6.604	6.777	-0.173
AA53	1273	489	6.1	6.34	-0.24
AA23	1559	549	5.791	5.821	-0.03
AA15	2576	555	4.334	4.537	-0.203

6. A note on 3-omega measurements on narrow (sub-micron) heaters

Supplementary figure 4 shows our experimental 3-omega data, Fourier law predictions and enhanced Fourier law predictions, with the latter's parameters derived from 2-omega experiments. We see that:

(a) The slopes of the graphs are identical beyond 200 Hz, and

(b) The discrepancies from the Fourier law are almost evenly split between non-Fourier effects and interfacial impedance effects. If the only information we had was the 3-omega experimental data and the Fourier law prediction, we would not been able to disambiguate the two effects.

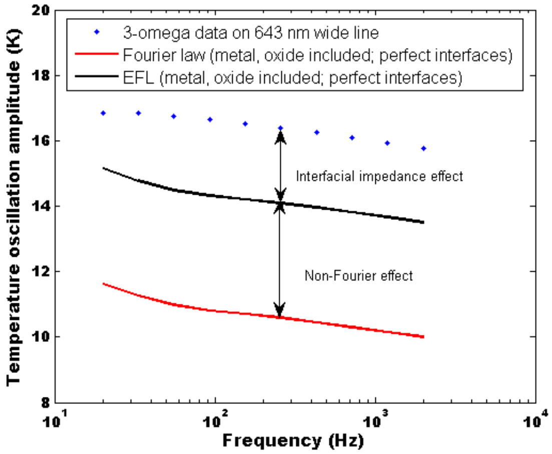

Supplementary Figure 4: From the 3-omega experiment alone, it is impossible to differentiate non-Fourier quasi-ballistic effects from interfacial impedance effects.

Here is where the 2-omega method is most useful. As we show in Fig. 5 of the main manuscript, the temperature measured at the thermometer is highly insensitive to the oxide, metal and interfacial resistances (±0.25% with or without).

This effect is best explained by an electrical analogy: in a two-probe electrical conductance measurement, where the voltage difference is measured across the same contact pads where current is sourced/sunk, the contact conductance contributes to the measured conductance and must be extracted out. In a four-probe measurement, where the voltage is measured across two other contact pads different from the ones where the current is sourced/sunk, the contact resistance does not influence the measured conductance.

References:

1. A. T. Ramu and J. E. Bowers, "On the solenoidal heat-flux in quasi-ballistic thermal conduction", *Journal of Applied Physics* 118, 125106 (2015)
2. Maznev, A. A., Jeremy A. Johnson, and Keith A. Nelson. "Onset of nondiffusive phonon transport in transient thermal grating decay." *Physical Review B* 84, no. 19 (2011): 195206.
3. A. T. Ramu and J. E. Bowers, "A generalized enhanced Fourier law and underlying connections to major frameworks for quasi-ballistic phonon transport". Available at http://arxiv.org/abs/1506.00668
